# Multiview state-of-health estimation for lithium-ion batteries using time–frequency image fusion and attention-based deep learning

**DOI:** 10.1371/journal.pone.0335351

**Published:** 2025-11-03

**Authors:** Peijian Jin, Shuo Yang, Xinwan Xu, Chaoqun Li, Shihao Guo, Wei Yan, Hui Miao, Shimei Sun

**Affiliations:** 1 School of Emergency Science and Engineering, Jilin Jianzhu University, Changchun, Jilin, China; 2 Jilin Provincial Key Laboratory of Fire Risk Prevention and Emergency Rescue for Building, Changchun, PR China; 3 Ji Lin Sinopoly New Energy Technology Co, Ltd., Liaoyuan, Jilin, China; The Hong Kong Polytechnic University, CHINA

## Abstract

Lithium-ion batteries are high-performance energy storage devices that have been widely used in a variety of applications. Accurate early-stage prediction of their remaining useful life is essential for preventing failures and mitigating safety risks. This study proposes a novel multiview approach for estimating the State-of-Health (SOH) of lithium-ion batteries by integrating time-domain and time–frequency features. Firstly, time-domain signals are transformed into time–frequency images using a wavelet transform. Three representative features are then selected and converted into grayscale images, which are combined into three-channel color images as inputs for a convolutional neural network (CNN) to extract spatial features. These features are subsequently passed into a long short-term memory (LSTM) network to capture spatial dependencies. In parallel, raw temporal features are processed through a two-stage attention mechanism to explore both temporal and spatial correlations, followed by another LSTM to model temporal dependencies. The outputs from the two branches are fused using weighted integration and passed through a fully connected layer to generate the final SOH estimate. Comparative experiments with four baseline models demonstrate that the proposed time–frequency fusion architecture significantly enhances prediction accuracy, and that each component makes a meaningful contribution to the overall performance.

## Introduction

Lithium-ion batteries (LIBs) are widely used as rechargeable power sources in daily applications such as drones, smartphones, electric vehicles, and satellites, owing to their advantages which include low weight, high energy and power density, wide operating range, and fast charging capability [1]. However, aging-related issues—particularly capacity degradation—can cause to significant performance decline and even catastrophic failures. Therefore, accurately predicting the State of Health (SOH) of lithium-ion batteries is critically important. Reliable SOH estimation provides valuable insights for product design enhancement, safety improvement, maintenance strategy optimization, and cost-benefit analysis. Currently, SOH estimation approaches are broadly classified into model-based methods and data-driven methods, with the latter primarily based on machine learning.

In the field of lithium-ion battery life prediction, international studies are broadly categorized into electrochemical model-based approaches. For instance, pseudo two-dimensional (P2D) models simulate capacity degradation by describing lithium-ion diffusion and reaction kinetics within electrodes [2]. Equivalent circuit models represent the dynamic behavior of batteries using resistor-capacitor networks, with parameters fitted from aging experiments [3]. Empirical degradation models, such as the Arrhenius equation, establish relationships between capacity fade and influencing factors like temperature or charge/discharge rates through accelerated aging experiments [4]. Collectively, these approaches characterize internal degradation processes from an electrochemical perspective. Wu et al. proposed a feature-enhanced encoder–decoder model that directly incorporates prior knowledge into measurement data to improve estimation accuracy. This method fuses key segments of the incremental capacity (IC) curve with voltage information, providing a novel strategy for battery SOH estimation [5].

In other research directions, Zou proposed a Bayesian model averaging-based SOH estimation method, which effectively accounts for both parameter and model uncertainties by integrating estimates from multiple models [6]. Luo introduced a physics-informed hybrid neural network that combines electrochemical, thermal, mechanical, and side reaction aging processes with data-driven modeling, resulting in more accurate predictions of battery capacity loss [7].

Recent studies have also explored the integration of feature engineering with machine learning. Wu selected health indicators exhibiting higher correlation with battery capacity by evaluating multiple features using Pearson correlation coefficients and scatter plots. The ReliefF algorithm was then applied for feature dimensionality reduction, followed by the construction of a convolutional neural network (CNN) for SOH estimation. The Kepler optimization algorithm was employed to fine-tune the model’s hyperparameters for optimal performance [8]. Yu extracted features from the battery’s incremental capacity (IC) curves and applied Gaussian filtering for denoising, selecting key health indicators. By innovatively combining spatiotemporal features with a CNN-MLP model, the proposed method achieved accurate SOH prediction, outperforming traditional models in both speed and accuracy [9].

Xian converted the current curves into differential current curves to extract key features, and utilized the Albatross optimization algorithm to optimize the parameters of a backpropagation neural network, leading to significant performance improvements [10]. Xu proposed an LSTM-based SOH estimation method incorporating data characteristics and a spatiotemporal attention mechanism. By distinguishing between trend and non-trend features and extracting multidimensional spatiotemporal information, the model demonstrated enhanced ability to capture dynamic behavior. Experimental results confirmed its superior accuracy and robustness [11].

Li introduced a method that analyzes the charging voltage curve to extract key health factors and computes battery health indicators closely related to aging. These features were then used to train and estimate battery SOH via machine learning models [12]. He investigated the influence of sampling frequency on SOH estimation accuracy, identifying the optimal frequency identifying the optimal frequency to maintain data integrity data integrity. The use of signal variance as a key feature enhanced the precision of health estimation [13].

In terms of neural network architecture innovation, Cui integrated convolutional neural networks with Arnold networks. The model incorporated learnable activation functions, and the proposed KANs (Kernel-based Adaptive Networks) addressed the limitations of traditional neural networks in flexibility [14].

In addition, multimodal data fusion approaches have received increasing attention in recent years. Chen proposed a multimodal fusion model that utilizes features extracted from voltage curves recorded during charging and histogram-based features captured during the battery aging process. This fusion strategy substantially enhanced the model’s generalization capability [15].

Furthermore, recent works such as MSRCN [16] utilize multi-scale residual convolutional networks to extract features from battery sequences, improving robustness under varying operating conditions, while M2BIST-SPNet [17] integrates multimodal inputs with spatiotemporal attention mechanisms, enhancing the detection of subtle degradation patterns. These studies are relevant to the multimodal feature-input approach proposed here. Despite numerous models proposed for lithium-ion battery state-of-health estimation, several common challenges remain:

1) Most existing feature extraction methods focus on parameters from both charging and discharging phases. However, due to significant individual differences in discharge strategies often lead to poor generalization of models trained on discharge data.
2) Traditional feature extraction mainly analyzes features in the time domain, with limited studies exploring multiview analysis that integrates both time-domain and frequency-domain representations.

To address these challenges, this study proposes a data-driven method that incorporating multiview features to enhance the model’s robustness, generalization, and estimation accuracy. The main contributions are as follows:

1) To enhance model generalization, this study utilizes data exclusively from the charging phase, ensuring more practical applicability.
2) Unlike conventional single time-domain features, this study employs multiview features by applying continuous wavelet transform to convert time-domain signals into frequency-domain image features. Subsequently, the remaining time-series features and frequency-domain features are trained separately to enhance the representation of battery degradation.

## Data processing

This section presents the aging test data of the selected batteries and explains the division of the dataset into training and testing sets. The preprocessed data are labeled based on the State of Health (SOH) to prepare them for subsequent input into the model for battery health assessment. The SOH is defined by the following formula:


$$SOH_{k}=\frac{C_{k}}{C_{\text{0}}}\times \text{1}\text{0}\text{0}\%$$

Here $C_{k}$ is the actual capacity of the battery at the current cycle, $C_{\text{0}}$ is the rated capacity of the battery at the initial state, and $SOH_{k}$ represents the state of health of the battery at the current cycle.

### Dataset description

**Fig 1:**
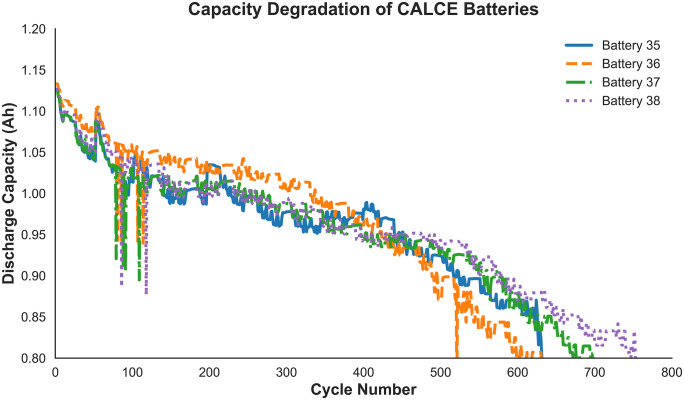
The capacity degradation curves for batteries in the dataset.

Data in this study were obtained from the Battery Lifecycle Engineering Center at the University of Maryland. Four LiCoO₂ batteries (CS2_35, CS2_36, CS2_37, and CS2_38) each with a rated capacity of 1.1 Ah were selected for analysis because they are the only batteries in the public dataset with complete and continuous charge–discharge records suitable for the proposed experiments. All batteries were tested at a constant temperature of 1°C. Charging was conducted in constant current (CC) mode up to 4.2 V, followed by constant voltage (CV) mode until the current decreased to 20 mA, and discharging was performed in CC mode until 2.7 V. The capacity degradation curves of the four batteries are shown in Fig 1.

### Feature extraction

Data in this study were limited to the battery’s charging phase, as discharge strategies are influenced by uncontrollable factors such as user-specific usage patterns and environmental loads. Charging typically occurs in stable conditions, making the data more reproducible and the charging strategy relatively consistent. Evaluating battery health based on charging features helps avoid biases caused by the randomness of discharging, and is therefore considered more representative of battery degradation.

#### Image feature selection.

If image features are directly selected from the current sequence of each cycle, the voltage and current curves under different aging states show little variation due to the constant current–constant voltage (CC-CV) charging strategy. Therefore, we normalize the current and voltage values using features that are more closely related to battery aging, specifically the constant current charging time and constant voltage charging time.

For image feature selection during the constant current charging phase, the constant current charging time divided by the charging current forms a time series that reflects battery aging, hereafter referred to as CIT-I. Similarly, during the constant voltage charging phase, the constant voltage charging time divided by the voltage at that moment forms another aging-related time series, referred to hereafter as CVT-V.

**Fig 2:**
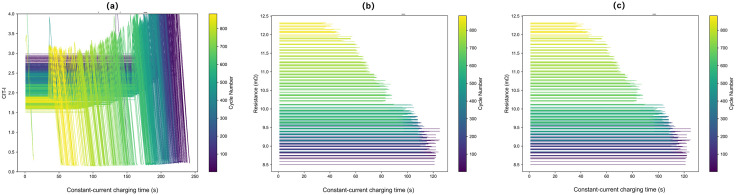
Variations of the selected features during battery aging. (a) CIT-I, the ratio of constant-current (CC) charging time to CC charging current (s/A), measured over successive cycles; (b) CVT-V, the ratio of constant-voltage (CV) charging time to the voltage at that moment (s/V), measured over successive cycles; (c) battery impedance (Ω) over the same cycles. Each curve corresponds to an individual battery cycle, illustrating the evolution of the feature values during the aging process.

This processing approach was chosen to preserve the physical significance of the charging process rather than applying simple mathematical transformations. Impedance was selected as the third feature, obtained directly from the University of Maryland battery dataset for each cycle of the CS2_35 battery, corresponding to the direct current internal resistance defined as the ratio of voltage change to applied current during constant current charging or discharging.The variations of these selected features during the battery aging process are shown in Fig 2(a)–(c).

The three selected features show distinct variations during battery aging. CIT-I gradually decreases, CVT-V gradually increases, and battery impedance rises due to factors such as the thickening of the solid electrolyte interphase (SEI) layer and loss of active materials. These feature sequences effectively characterize the battery’s state of health at different degradation levels.

#### Electrical parameter feature selection.

To deeply analyze the electrochemical behavior of the battery during charge and discharge processes, incremental capacity (IC) analysis is performed by differentiating the voltage–capacity curve. The IC curve represents the first derivative of capacity with respect to voltage, as shown in Equation 1.


$$\frac{dQ}{dV}\approx \frac{Q_{i+\text{1}}-Q_{i}}{V_{i+\text{1}}-V_{i}}$$ (1)

$\frac{dQ}{dV}$ represents the rate of change of capacity per unit voltage; $Q_{i}$ denotes the capacity corresponding to the iii-th voltage point; $V_{i}$ denotes the voltage at the iii-th sampling point; and $Q_{i+\text{1}}$ denotes the capacity corresponding to the (i+1)-th voltage point.

**Fig 3:**
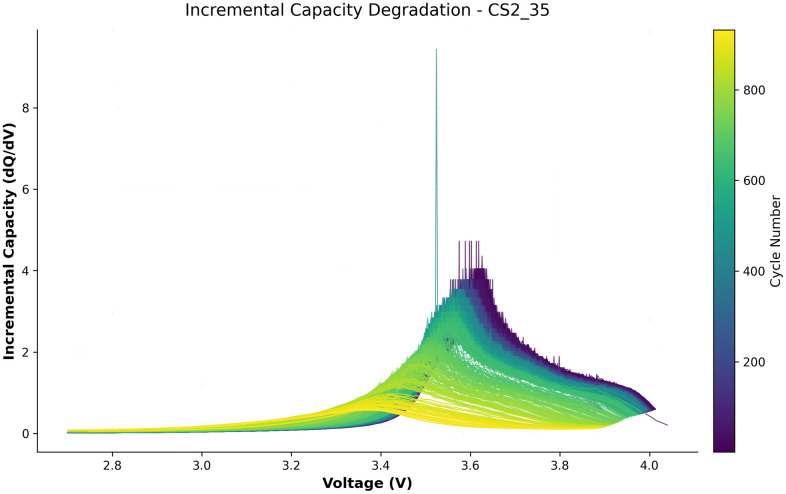
Incremental capacity (IC) curves during the battery aging process.

$V_{i+\text{1}}$ denotes the voltage at the (i+1)-th sampling point. The incremental capacity (IC) curve for battery CS2_35 is shown in Fig 3 as an example.

As the battery undergoes cyclic aging, its coulombic efficiency and the peak of the incremental capacity (IC) curve gradually decline. The IC peak thus serves as a key parameter reflecting the battery’s aging state, closely related to mechanisms such as loss of lithium and electrode material degradation.

**Fig 4:**
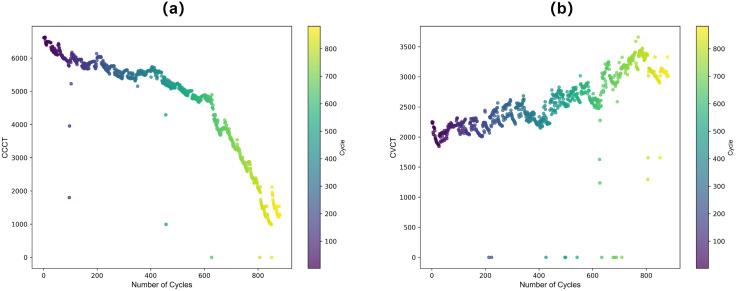
(a)-(b) The CCCT and CVCT curves during the battery aging process.

To comprehensively assess battery aging, two additional features were used: constant current charging time (CCCT) and constant voltage charging time (CVCT), as shown in Fig 4a–b. These features vary significantly under different aging conditions and were analyzed in both time and frequency domains. Time-domain analysis captures the overall degradation trend, while frequency-domain analysis highlights oscillations, nonlinearities, and subtle fluctuations, improving the model’s performance.

By fusing these features, a comprehensive quantitative assessment of battery aging is achieved, providing robust input for lifetime-prediction models.

## Materials and methods

**Fig 5:**
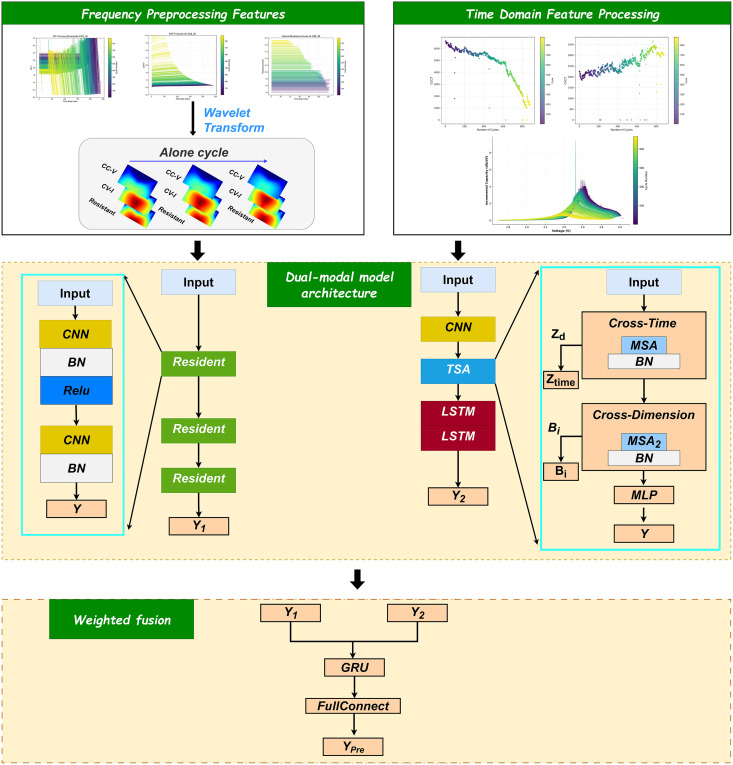
Overall flowchart of the proposed methodology.

In this section, we present the construction of a combined frequency-domain and time-domain neural network. The flowchart of the proposed algorithm is shown in Fig 5. First, three features—constant current charging time divided by the constant current (CIT-I), constant voltage charging time divided by the constant voltage (CVT-V), and impedance—are transformed via wavelet transform into three-channell image features. A convolutional neural network (CNN) is then applied to extract spatiotemporal features of individual cycles, while a long short-term memory network (LSTM) is used to capture temporal dependencies.

Next, a two-stage temporal-spatial attention (TSA) mechanism is employed to extract temporal and spatial features from the time-domain data. The outputs from both time-domain and frequency-domain branches are concatenated and fed into a gated recurrent unit (GRU) network, which dynamically allocates weights between the two modalities before producing the final state-of-health (SOH) estimate for the lithium-ion battery.

### Wavelet transform

Wavelet transform is a widely used mathematical tool in the time-frequency domain [18]. Traditional Fourier transform only provides the overall frequency information of a signal and cannot reveal temporal variations in frequency. This limitation makes it insufficient for analyzing the dynamic aging process of batteries. In contrast, wavelet transform decomposes the signal into wavelet functions at different scales and positions, overcoming the limitations of Fourier transform and making it suitable for processing non-stationary battery signals. The mother wavelet selected in this study is the Mexican Hat wavelet, which is particularly appropriate for nonlinear data. The wavelet transform is defined as shown in Equation 2:


$$W_{\psi }(a,b)=\frac{\text{1}}{\sqrt{\left|a\right|}}\int_{-\infty }^{+\infty }\;x(t)\;\psi^{*}\left(\frac{t-b}{a}\right)dt\;$$ (2)

where x(t)is the original signal, ψ() is the mother wavelet function, $\psi^{*}()$ denotes the complex conjugate of the mother wavelet, a is the scale factor (controlling frequency), and b is the translation factor (controlling time position).

**Fig 6:**
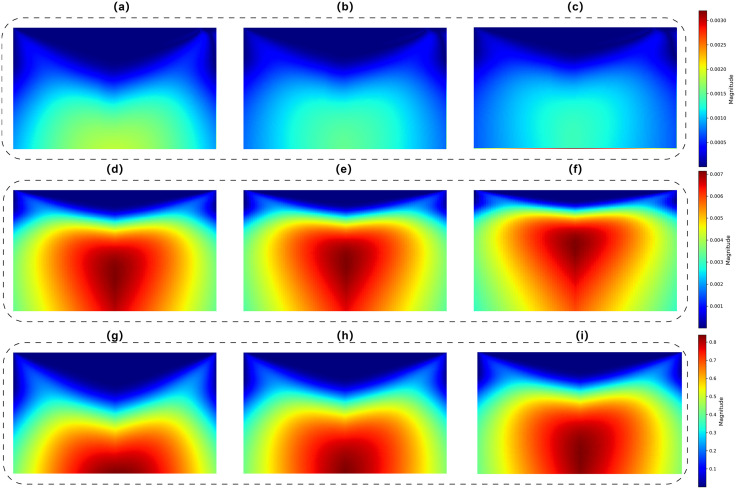
Figs (a)–(i) show the different stage images after wavelet transformation. (a)–(c) correspond to the early, middle, and late stages images of CIT-I; (d)–(f) correspond to the early, middle, and late stages images of CVT-V; (g)–(i) correspond to the early, middle, and late stages images of impedance.

The wavelet-transformed images better reveal the energy variations of the battery across different frequency bands. As the battery ages, high-frequency components gradually increase. After transformation into two-dimensional images, these features are more easily recognized by visual models compared to the original time series. In this study, the CWT was performed using the Ricker wavelet (Mexican Hat) with scales ranging from 1 to 128. The resulting coefficients were mapped into 32 × 32 images for input into the frequency-domain model. The Mexican Hat wavelet was chosen as the mother wavelet due to its enhanced suitability for nonlinear data, as it effectively captures transient changes and local anomalies in the battery signals, which are highly correlated with battery aging mechanisms. The wavelet-transformed images of the time series are shown in Fig 6, representing the CS2_35 battery at the early, middle, and late stages of aging, respectively.

Distinctive feature maps are observed at different stages of battery aging, indicating that wavelet transform can be effectively used to extract characteristic images for estimating battery health. To facilitate model input when processing two-dimensional images, the grayscale wavelet-transformed feature maps are stacked to create three channels, forming a color image. These fused color image features are then fed into the convolutional neural network.

### Convolutional neural network

**Fig 7:**
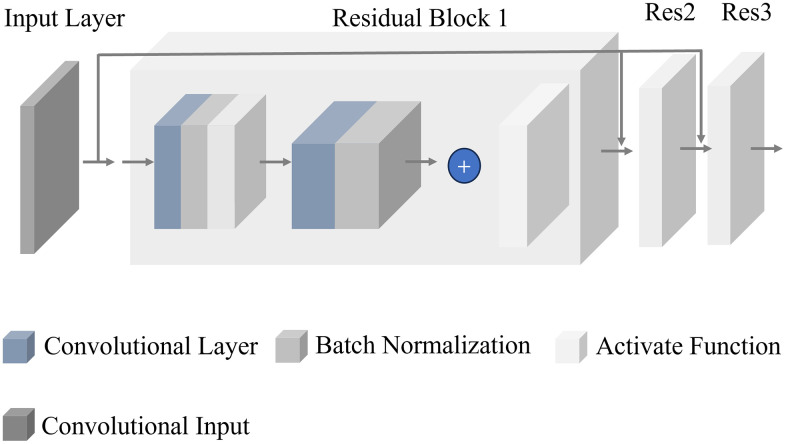
CNN picture extractor.

When image data and time-series data form a one-dimensional grid, convolutional neural networks (CNNs) demonstrate strong capabilities in processing such structures [19]. CNN data contain multiple channels, where each channel may correspond to the three channels of an RGB color image or different variables along the time axis. Observing variables along the time axis is defined as a one-dimensional convolutional neural network (1D-CNN). This model can effectively capture variations in electrical parameters caused by battery aging. Fig 7 illustrates the architecture of the model used for image feature extraction.

In this study, the CNN feature extractor consists of four residual blocks and an adaptive max pooling layer, as shown in the figure. To ensure training stability and prevent overfitting, batch normalization layers and the ReLU activation function are incorporated into the network.

The input consists of two-dimensional images obtained from wavelet transform, containing characteristic information of the battery at different aging stages. The image data first pass through a convolutional layer for initial feature extraction, capturing spatial features within the images. This process is described by Equation 3:


x(con1,out)=x(con1,in)⊙W(con1) (3)

Where, ⊙ denotes the element-wise multiplication operation. x(con1,in) and W(con1) represent the input and weights of the first convolutional layer, respectively. To ensure training stability and prevent issues such as vanishing gradients, the output of the convolutional layer is fed into a normalization layer, as shown in Equation 4:


$$x(\text{BN}\text{1},\text{out})=\gamma ⊙\frac{x(\text{BN}\text{1},\text{in})-{\hat{\mu }}_{B}}{{\hat{\sigma }}_{B}+\in }+\beta$$ (4)

x(BN1,in) is the input to the normalization layer; γ and β are the learnable scaling and shifting parameters, respectively; ${\hat{\sigma }}_{B}$ and ${\hat{\mu }}_{B}$ are the standard deviation and mean of the current mini-batch features; and ∈ is a small constant added to avoid division by zero.xBN1,out is the output after batch normalization, as shown in Equations 5 and 6:


x(Relu,out)=σ(x(BN1,out)) (5)

y=x(conv1,out)=x(relu,out)⊙W(conv2) (6)

Here, σ denotes the activation function, which is chosen as ReLU. ReLU enables the neural network to learn and represent more complex patterns and helps alleviate the vanishing gradient problem. x(Relu,out) is the output after the activation function. W(conv2) represents the weights of the second convolutional layer, and y denotes the output of the first residual block.

### Long short-term memory (LSTM) neural network

**Fig 8:**
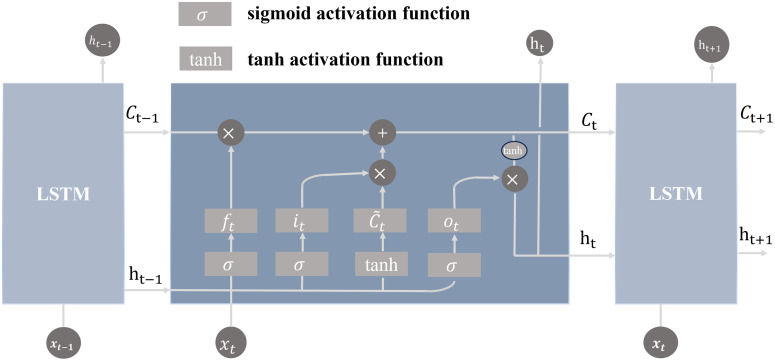
LSTM architecture.

Battery aging occurs over hundreds to thousands of cycles, with varying degrees of correlation between degradation information from different cycles. To efficiently extract these degradation features, traditional neural networks such as GRU and RNN face challenges like gradient explosion or vanishing gradients when dealing with long time series. These issues hinder the accurate estimation of the battery’s remaining useful life. For long-term sequence prediction tasks like battery life estimation, Long Short-Term Memory (LSTM) networks are particularly suitable. The structure of an LSTM cell is illustrated in Fig 8.

At this stage, the LSTM processes the battery’s electrical parameter data along the time dimension. The mathematical expression of the forget gate ftf_tft is given by Equation 7:


$$f_{t}=\sigma (W_{f}\cdot [h_{t-\text{1}},N_{t}]+b_{f})$$ (7)

The mathematical expression of the input gate $i_{t}$ is given by Equation 8:


$$i_{t}=\sigma (W_{i}\cdot [h_{t-\text{1}},N_{t}]+b_{i})$$ (8)

The mathematical expression of the candidate memory cell ${\tilde{c}}_{t}$ is given by Equation 9:


$${\tilde{c}}_{t}=\text{tanh}\left(W_{c}\cdot [h_{t-\text{1}},N_{t}]+b_{c}\right)$$ (9)

The mathematical expression of the cell state $c_{t}$ is given by Equation 10:


$$c_{t}=f_{t}⊙c_{t-\text{1}}+i_{t}⊙{\tilde{c}}_{t}$$ (10)

The mathematical expression of the output gate $o_{t}\;$is given by Equation 11:


$$o_{t}=\sigma (W_{o}\cdot [h_{t-\text{1}},N_{t}]+b_{o})$$ (11)

The mathematical expression of the hidden state $h_{t}$is given by Equation 12:


$$h_{t}=o_{t}⊙\text{tanh}\left(c_{t}\right)$$ (12)

The mathematical expression of the total output of the LSTM layer, $H_{i}$ is given by Equation 13:


$$H_{i}=\text{LSTM}(X_{i}^{\prime })$$ (13)

### Two-stage attention mechanism

**Fig 9:**
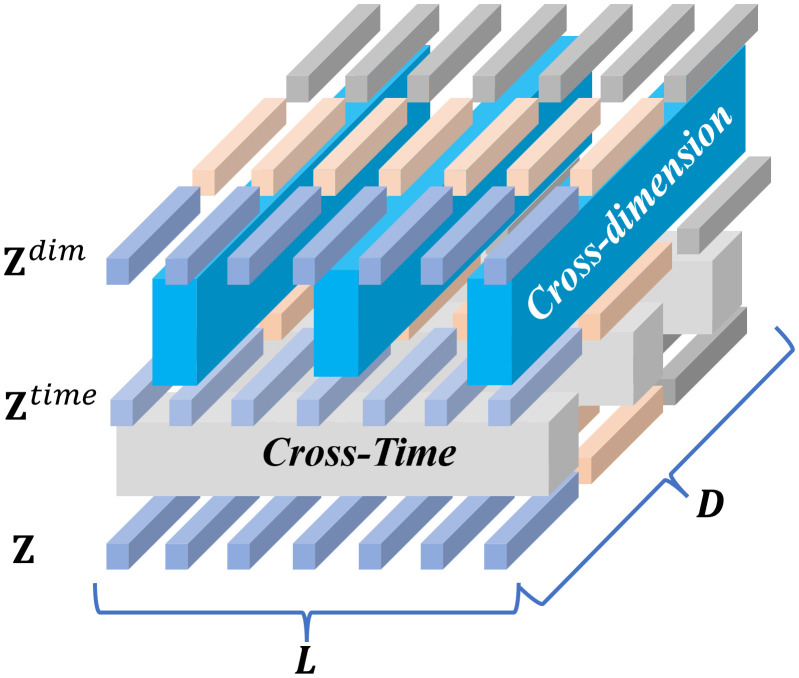
Two-stage attention layer process.

Traditional attention mechanisms often consider only relationships along the temporal dimension or among different feature channels independently. In this study, the attention mechanism is divided into two stages, which differing from conventional approaches. Since the time axis and the feature dimension axis of multivariate time series represent different meanings, the attention mechanism is separated accordingly into two stages. The two-stage attention framework is illustrated in Fig 9.

The two-stage attention layer (TSA) is designed to process a two-dimensional vector array representing multivariate time series, where each vector corresponds to a segment of the original sequence. The entire vector array undergoes two sequential stages to capture the respective dependencies: the first stage is the cross-temporal attention mechanism, which applies multi-head attention separately to each feature dimension. The expressions are given in Equations 14 and 15.


$${\hat{𝐙}}_{:,d}^{time}=\text{LNorm}\left({𝐙}_{:,d}+{\text{MSA}}^{time}\left({𝐙}_{:,d},{𝐙}_{:,d},{𝐙}_{:,d}\right)\right)$$ (14)

$${𝐙}^{time}=\text{LNorm}\left({\hat{𝐙}}^{time}+\text{MLP}\left({\hat{𝐙}}^{time}\right)\right)$$ (15)

Here, LNorm denotes layer normalization, and MLP represents a multilayer perceptron network. MSA(Q,K,V) refers to the multi-head self-attention layer with queries Q, keys K, and values V. All feature dimensions share the same MSA layer. After capturing temporal dependencies through MSA and MLP, the output ${𝐙}^{time}$ is obtained. This output then serves as the input for the second stage, which captures spatial dependencies.

**Fig 10:**
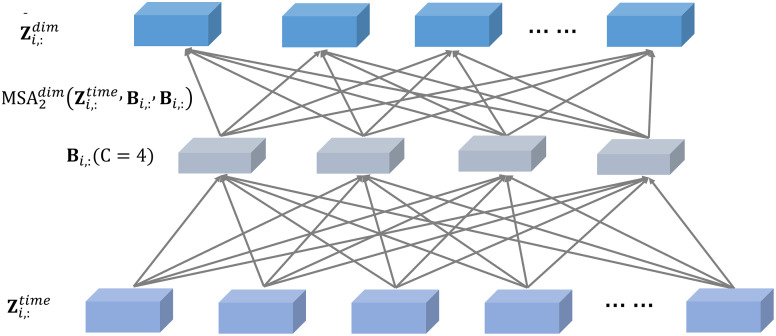
Across dimension attention.


In the cross-dimension stage, when the data dimension is very large, directly applying the multi-head self-attention (MSA) leads to a dramatic increase in computational complexity. Therefore, an intermediate representation mechanism is introduced, as illustrated in Fig 10. The number of intermediate vectors is denoted as CCC. This mechanism serves two main purposes: using the intermediate vectors as queries, it treats all dimension vectors as keys and values, and employs multi-head attention to aggregate global dimensional information. The expression is given by Equation 16.

$${𝐁}_{i,:}={\text{MSA}}_{\text{1}}^{dim}({𝐑}_{i,:},{𝐙}_{i,:}^{time},{𝐙}_{i,:}^{time}),\text{1}\leq i\leq L$$ (16)

${𝐑}_{i,:}$denotes the learnable intermediate information array, and ${𝐁}_{i,:}$ represents the aggregated information among dimensions after processing. Then, using the dimensions as queries and the aggregated intermediate information as keys and values, the information is redistributed to all global dimensions.


$${\overset{¯}{𝐙}}_{i,:}^{dim}={\text{MSA}}_{\text{2}}^{dim}({𝐙}_{i,:}^{time},{𝐁}_{i,:},{𝐁}_{i,:})$$

After obtaining the outputs from the cross-temporal and cross-dimensional channels, the two-stage outputs are integrated. The expressions are given by Equations 17 and 18:


$${\hat{𝐙}}^{dim}=\text{LayerNorm}\left({𝐙}^{time}+{\bar{𝐙}}^{dim}\right)$$ (17)

$${𝐘=𝐙}^{dim}=\text{LayerNorm}\left({\hat{𝐙}}^{dim}+\text{MLP}({\hat{𝐙}}^{dim})\right)$$ (18)

## Experimental results

This section primarily discusses the performance of the proposed model in lithium-ion battery health assessment. Based on ablation experiments, different functional modules are replaced or disabled to verify the necessity of each component.

### Experimental conditions and setup

The proposed multimodal battery health assessment model and other comparison models were tested on a computer running Ubuntu 20.04, equipped with an Intel(R) Xeon(R) Platinum 8255C processor and an RTX 4050 GPU. All algorithms were implemented using Python 3.8 and the PyTorch 2.0 framework. The overall model training employed the Adam optimizer. The error metrics used were Mean Absolute Error (MAE), Mean Absolute Percentage Error (MAPE), Root Mean Square Percentage Error (RMSPE), and Root Mean Square Error (RMSE), with their respective formulas shown in Equations 19–22:


$$\text{MAE}=\frac{\text{1}}{n}\sum_{i=\text{1}}^{n}\;|y_{i}-{\hat{y}}_{i}|$$ (19)

$$MAPE=\frac{\text{1}\text{0}\text{0}\%}{N}\sum_{i=\text{1}}^{N}\;\left|\frac{y_{i}-{\hat{y}}_{i}}{y_{i}}\right|$$ (20)

$$RMSPE=\sqrt{\frac{\text{1}\text{0}\text{0}\%}{N}\sum_{i=\text{1}}^{N}\;{\left(\frac{y_{i}-{\hat{y}}_{i}}{y_{i}}\right)}^{\text{2}}}$$ (21)

$$RMSE=\sqrt{\frac{\text{1}}{N}\sum_{i=\text{1}}^{N}\;{\left(y_{i}-{\hat{y}}_{i}\right)}^{\text{2}}}$$ (22)

Generally, appropriate hyperparameter selection ensures a good balance between model generalization, training speed, and stability. In this study, the hyperparameters were chosen as follows: the size of the wavelet-transformed images is 32 × 32. The model processing the frequency-domain data consists of a single convolutional layer followed by two LSTM layers, each with 128 hidden units. This is followed by a fully connected layer with ReLU activation and another fully connected layer that outputs the frequency-domain prediction results. Dropout rates of 0.5 and 0.3 are applied after the LSTM layers and activation function, respectively. For the time-domain data model, two convolutional layers are followed by a TSA-LSTM module, where the TSA module uses 4 attention heads, and each LSTM layer has 64 hidden units. The sliding window stride for constructing time-series subsequences is set to 1 cycle. The batch size is set to 64, the number of epochs is 40, and the learning rate is 0.001. The loss function used for training is Mean Squared Error (MSE).

In this study, the leave-one-out (LOO) strategy was employed for model evaluation. Specifically, the four battery datasets were alternately designated as the test set, while the remaining three served as the training set for model development and validation. This cross-validation approach enables a comprehensive assessment of the model’s generalization ability and robustness across different batteries, thereby avoiding biases introduced by a single test split and ensuring the reliability and objectivity of the evaluation. In each round, the model parameters were thoroughly optimized on the training set, and prediction error metrics were computed on the test set to quantify performance. The results from all rounds were then aggregated to provide an overall evaluation of the model.

### Hyperparameter selection

#### Sliding window.

**Fig 11:**
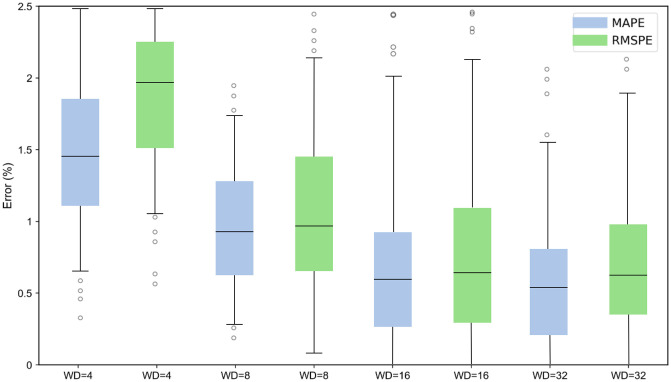
Prediction errors for different sliding window sizes. The horizontal axis represents the sliding window size (WD = 4, 8, 16, 32), and the vertical axis shows the error values. Two error metrics are presented: mean absolute percentage error (MAPE) and root mean square error (RMSE), as indicated in the legend..

To fully exploit the temporal dependency features in battery time series data, this study employs a sliding window strategy to reconstruct the original sequence. The sliding window method divides continuous time series into multiple overlapping subsequences, providing local contextual information to the model and enhancing prediction performance. In this study, window lengths of 4, 8, 16, and 32 are compared. By sliding the window along the time axis with a fixed step size, the model is trained and evaluated under different window settings to determine the optimal configuration. Taking battery CS_35 as an example, the prediction errors corresponding to different window sizes are shown in Fig 11, and detailed in Table 1. As observed from the error plots, when the window size is set to 16, the model achieves the lowest RMSE of 0.59%. Increasing the window size to 32 yields no significant improvement. Therefore, a window size of 16 is used in the subsequent experiments.

**Table 1:** Comparison of error metrics under different sliding window sizes for cell CS_35.

Window size	MAE(Ah)	RMSE(%)	MAPE(%)
4	0.00697	1.88	1.43
8	0.00351	0.84	0.74
16	0.00197	0.59	0.53
32	0.00184	0.47	0.41

#### Selection of the number of intermediate units in the attention mechanism.

**Fig 12:**
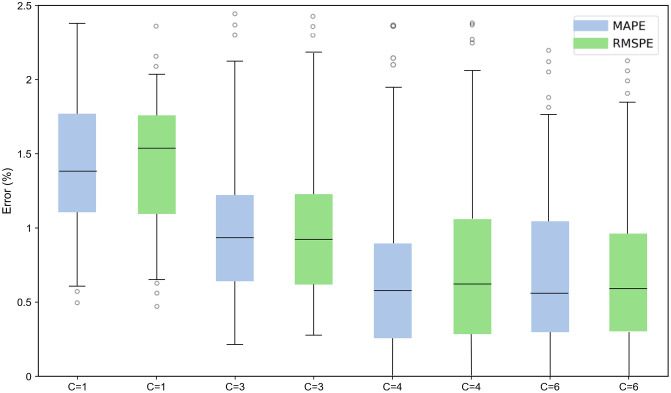
Errors under different quantities of intermediate information: C = 1、C = 3、C = 4 and C = 6.

In the memory-based attention mechanism used in this study, the number of intermediate representations has a significant impact on both the training speed and prediction accuracy of the model. To determine the optimal number of intermediate units, various configurations were tested to evaluate their influence on overall model performance. With the sliding window size fixed at 16 and using battery CS_35 as an example, Fig 12 shows that after the number of intermediate units reaches 4, further increases lead to slight improvements in prediction accuracy but at the cost of significantly higher computational complexity. Therefore, this study selects 4 as the number of intermediate representations. The corresponding prediction errors for different numbers of intermediate units are presented in Table 2.

**Table 2:** Comparison of error metrics with different numbers of intermediate information groups for cell CS_35.

C	MAE(Ah)	RMSE(%)	MAPE(%)
1	0.00917	1.58%	1.49%
2	0.00197	0.91%	0.90%
4	0.00397	0.78%	0.72%
6	0.00450	0.77%	0.71%

### Ablation study and model validation of multiview inputs

To ensure that each module in the proposed model contributes positively to the battery state-of-health estimation, ablation experiments were conducted on three components: image feature extraction, time-series feature extraction, and the TSA attention mechanism. The wavelet-transformed images enhance data dimensionality, while time-series feature extraction follows a conventional approach. The TSA attention mechanism captures dependencies in both the temporal and spatial dimensions. To validate the effectiveness of integrating these three components, ablation studies were performed.

**Fig 13:**
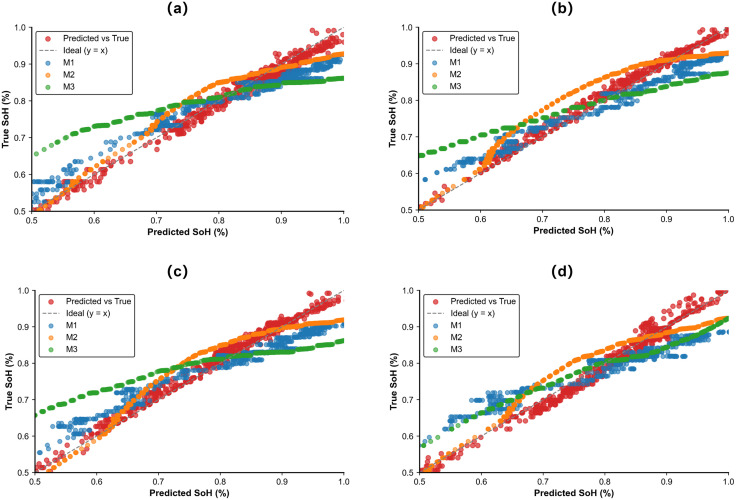
(a)- (d) Results of the ablation study for each Battery on CS2_35, CS2_36, CS2_37 and CS2_38.

During the testing phase, three evaluation metrics were used to assess the model’s prediction accuracy. The training process adopted the leave-one-out strategy, where three out of the four batteries were used for training, and the remaining one was used for validation, repeated across four rounds. Table 3 presents the results of the ablation experiments on different datasets. Fig 13 compares the prediction errors in bar chart form under a sliding window size of 16 cycles. Specifically, M1 represents the full model structure; M2 uses only time-domain data for prediction; M3 uses only frequency-domain data; and M4 is the model with the TSA module removed.

**Table 3:** Comparison of error results in ablation experiments.

Indicators	Models	CS_35	CS_36	CS_37	CS_38
MAE(Ah)	M1	0.00197	0.00168	0.00151	0.00231
	M2	0.00951	0.00992	0.00864	0.00955
	M3	0.00864	0.00764	0.00721	0.00903
	M4	0.01264	0.02011	0.00919	0.01401
RMSE(%)	M1	0.48%	0.62%	0.52%	0.66%
	M2	1.44%	2.55%	2.81%	2.13%
	M3	1.36%	2.23%	2.10%	2.30%
	M4	1.88%	2.60%	2.20%	2.40%
MASPE(%)	M1	0.43%	0.75%	0.65%	0.73%
	M2	1.34%	2.01%	2.79%	1.96%
	M3	1.21%	2.11%	2.03%	1.91%
	M4	1.68%	2.33%	2.13%	2.11%

From the analysis of the results, it can be observed that the model incorporating wavelet-transformed features achieves lower prediction error compared to the model using only one-dimensional time-series data. The inclusion of the TSA attention mechanism significantly improves the prediction accuracy over the time-series model without attention. Moreover, the overall model that integrates both two-dimensional image features and one-dimensional time-series features processed by the TSA mechanism yields lower prediction errors than those using each module individually.

**Fig 14:**
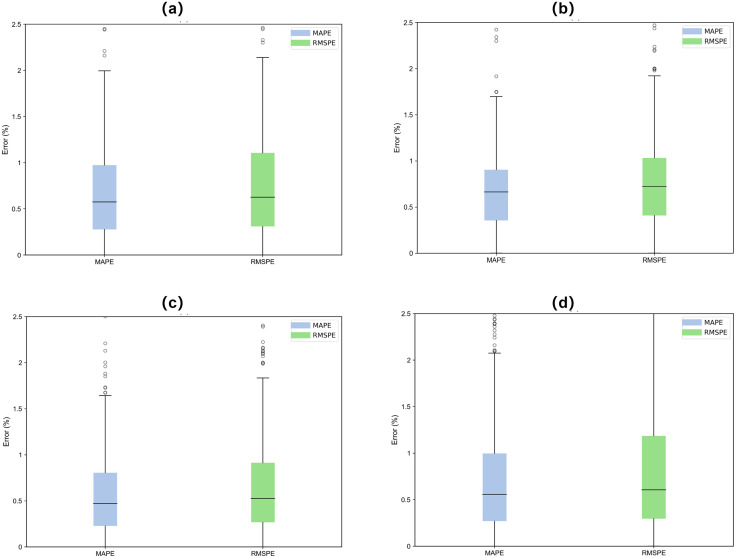
(a)–(d) Estimation error distribution in each battery.

Fig 14 illustrates the distribution of evaluation errors for each aging cycle of each battery. The results indicate that the proposed model provides relatively stable state-of-health estimations throughout the entire battery aging process, with RMSE values consistently below 2%.

### Comparison of evaluation results with other models

To verify the accuracy of the proposed method for battery state-of-health estimation, we compare its performance with other models. Model A (VMD-BiLSTM-BO [20]) employs Variational Mode Decomposition (VMD) to perform multi-scale decomposition of the raw signals, extracting features from different frequency bands. It combines a Bidirectional Long Short-Term Memory network (BiLSTM) to capture temporal dependencies and uses Bayesian Optimization (BO) to automatically tune hyperparameters, achieving efficient battery life prediction. Model B (Explainability-driven LSTM-CNN [21]) integrates Long Short-Term Memory (LSTM) and Convolutional Neural Networks (CNN) to extract temporal and local spatial features through deep learning, incorporating explainability mechanisms to enhance the model’s focus on key features and improve prediction transparency and accuracy. Model C (GBDT+SSA + FFT [22]) utilizes Gradient Boosting Decision Trees (GBDT) as the main predictor, combined with Spatial Sparse Attention (SSA) for optimized feature selection, and Fast Fourier Transform (FFT) to extract frequency-domain features, effectively modeling battery performance degradation by leveraging both feature engineering and machine learning advantages. In the figure, the X-axis represents the battery cycle number, and the Y-axis indicates the battery capacity values predicted by the models.

All three baseline models were originally evaluated on the same battery dataset (CS2_35–CS2_38) used here. In this work, their reported results are directly cited from the original publications without retraining or modification. This ensures the use of validated code and hyperparameters while maintaining transparency. However, as these models were not re-run under the present experimental pipeline, minor differences in implementation, data preprocessing, or random seeds may exist. Therefore, their metrics provide a reference for comparison but are not strictly comparable to the results of the proposed model.

To ensure robustness, each experiment with the proposed model was repeated five times, and the results are reported as mean ± standard deviation. The prediction results demonstrate that the multiview feature input model can accurately estimate the available battery capacity, with minimal error between the estimated and actual values. Compared to other algorithms, the inclusion of two-dimensional image features significantly improves the model’s prediction accuracy. In contrast, using only one-dimensional time-series features shows limited improvement once a certain accuracy level is reached. Detailed error metrics for each model are presented in Table 4.

**Table 4:** Comparison of error metrics among different models.

Indicators	Models	CS_35	CS_36	CS_37	CS_38
MAE(Ah)	ours	0.00197 ± 0.00015	0.00168 ± 0.00014	0.00151 ± 0.00013	0.00231 ± 0.00016
	Model A	–	0.01600	0.24500	–
	Model B	0.01012	0.01650	0.016500	0.09460
	Model C	0.01620	0.01140	0.01040	0.01570
MAPE(%)	ours	0.00531 ± 0.00051	0.00583 ± 0.00056	0.00482 ± 0.00064	0.00541 ± 0.00071
	Model A	–	–	0.00480	–
	Model B	–	–	–	–
	Model C	–	–	–	–
MASPE(%)	ours	0.00431 ± 0.00081	0.00753 ± 0.00074	0.00654 ± 0.00043	0.00732 ± 0.00085
	Model A	–	0.01900	0.04800	–
	Model B	0.01250	0.02220	0.01980	0.09790
	Model C	0.01480	0.02850	0.01170	0.01410

^a^Missing values (“–”) indicate metrics that were not reported in the corresponding references.

As shown in Table 4, the multiview input model achieves overall high prediction accuracy across the four battery datasets. Among them, the prediction accuracy for Battery_37 is the highest, with RMSE and MAE values of 0.00151 Ah and RMSPE of 0.0052. For Battery_38, relatively large errors occur in the early aging cycles; however, as the aging process progresses, the prediction results gradually improve, with MAE and RMSPE decreasing to 0.00231 Ah and 0.0058, respectively. Furthermore, the battery capacity degradation curve reveals a noticeable capacity regeneration phenomenon during aging, which is well captured by the multiview feature input model.

## Conclusion

Accurately assessing the state-of-health (SOH) of lithium-ion batteries is crucial for quality evaluation and timely fault warning during battery aging. In this study, we utilized the first 16 charging cycles’ data of the battery, extracting features from wavelet-transformed images and one-dimensional electrical parameter time series. These features were processed respectively through CNN-LSTM and TSA-LSTM model frameworks to capture effective frequency-domain and time-domain characteristics. A series of experiments were conducted to evaluate the contribution of each network module to the overall model performance, and the proposed method was compared with existing battery health assessment models. The main conclusions are summarized as follows:

1. By reconstructing the electrical parameter features, continuous wavelet transform was applied to the constant current charging time divided by the constant current, the constant voltage charging time divided by the constant voltage, and impedance data to obtain frequency-domain feature images. These frequency-domain features capture information that is lacking in the time domain.
2. Compared with traditional attention mechanisms, the Two-Stage Attention (TSA) mechanism simultaneously considers dependencies across the temporal dimension and different feature channels, enabling the model to capture interdependencies in multidimensional time series and thus enhancing prediction accuracy.
3. Previous battery health assessment models typically use either frequency-domain or time-domain data independently as input. In contrast, this study integrates time-domain and frequency-domain models, resulting in feature selection that better represents battery degradation characteristics and consequently yields more accurate prediction results.

This research highlights the significance of multiview feature inputs for battery health estimation. Since the current experimental dataset does not include the critical temperature feature, future work will incorporate temperature-related correlations to further improve the prediction accuracy of the model.
